# Urinary Excretion of Neutrophil Gelatinase-Associated Lipocalin in Diabetic Rats

**DOI:** 10.1155/2014/961326

**Published:** 2014-08-27

**Authors:** Abraham Said Arellano-Buendía, Fernando Enrique García-Arroyo, Magdalena Cristóbal-García, María Lilia Loredo-Mendoza, Edilia Tapia-Rodríguez, Laura Gabriela Sánchez-Lozada, Horacio Osorio-Alonso

**Affiliations:** ^1^Renal Pathophysiology Laboratory, Department of Nephrology, Instituto Nacional de Cardiología “Ignacio Chávez”, Juan Badiano 1, Sección XVI, Tlalpan, 14080 México City, DF, Mexico; ^2^Histopathology Laboratory, Research Subdivision, School of Medicine, Universidad Panamericana, Donatello 43, 03910 México City, DF, Mexico

## Abstract

Recent studies suggest that tubular damage precedes glomerular damage in the progression of diabetic nephropathy. Therefore, we evaluated oxidative stress and urinary excretion of tubular proteins as markers of tubular dysfunction. *Methods.* Diabetes was induced in rats by streptozotocin administration (50 mg/kg). Oxidative stress was assessed by measuring the activity of catalase (CAT), glutathione peroxidase (GPx), and superoxide dismutase (SOD); additionally, expression levels of 3-nitrotyrosine (3-NT), 4-hydroxynonenal (4-HNE), and oxidized protein (OP) were quantified. Whole glomerular filtration rate (GFR) was measured. Urinary excretion of neutrophil gelatinase-associated lipocalin (uNGAL), osteopontin (uOPN), and N-acetyl-*β*-D-glucosaminidase (uNAG) was also determined. *Results.* Diabetic rats showed an increase in uNGAL excretion 7 days following induction of diabetes. Diuresis, proteinuria, albuminuria, creatinine clearance, and GFR were significantly increased by 30 days after induction. Furthermore, there was an increase in both CAT and SOD activity, in addition to 3-NT, 4-HNE, and OP expression levels. However, GPx activity was lower. Serum levels of NGAL and OPN, as well as excretion levels of uNGAL, uOPN, and uNAG, were increased in diabetics. Tubular damage was observed by 7 days after diabetes induction and was further aggravated by 30 days after induction. *Conclusion.* The tubular dysfunction evidenced by urinary excretion of NGAL precedes oxidative stress during diabetes.

## 1. Introduction

Diabetic nephropathy (DN) is now the primary cause of end stage renal disease (ESRD), described as a worldwide medical catastrophe. Although several lines of evidence have suggested that poor glycemic control undoubtedly plays a significant role [1, 2], the metabolic events responsible for triggering DN are not well understood.

During diabetes, persistent hyperglycemia through glucose autoxidation and defective antioxidant defenses causes increased production of reactive oxygen species (ROS) [3, 4]. This excessive ROS production and subsequent oxidative damage have been suggested as common outcomes of diabetes, which finally culminates in DN [4].

The impairment of renal function in DN patients has long been diagnosed using biochemistry tools, including measurement of serum creatinine (SCr) and blood urea nitrogen (BUN). However, these are not reliable markers of early loss of renal function, thus delaying therapeutic interventions in order to stop or slow progression of renal damage. Microalbuminuria has recently emerged as a sensitive marker of early renal damage; however, this measurement also lacks the sensitivity to detect the earliest changes in renal function.

Renal damage markers, particularly those associated with tubular dysfunction such as neutrophil gelatinase-associated lipocalin (NGAL), N-acetyl-*β*-D-glucosaminidase (NAG), and kidney injury molecule (KIM-1), have recently attracted attention as sensitive and specific biomarkers to detect early kidney damage [5, 6]. Extensive reports have verified the prognostic and diagnostic value of these markers in various renal disorders, including DN [5–7].

NGAL is a member of the lipocalin family originally identified as a 25-kDa protein covalently associated with human matrix metalloproteinase 9 (MMP-9) in human neutrophils [8]. It is primarily stored in specific granules of neutrophils but also expressed at very low levels in the kidney, trachea, lungs, stomach, and colon [9]. NGAL has diverse functions, including transporting and activating MMP-9, inducing apoptosis, and regulating the immune response. In pathological processes, evidence suggests that NGAL is tightly associated with a series of renal dysfunctions. NGAL is one of the most robustly expressed proteins in ischemic or nephrotoxic kidney injury in experimental models [10] and in humans [11]. Serum NGAL has been described as a sensitive and specific biomarker for early identification of acute kidney injury following cardiac surgery [11] and a novel biomarker in children with chronic kidney diseases [12].

Because diabetic nephropathy represents one of the most devastating outcomes during the progression of diabetes mellitus, early detection strategies to diagnose loss of renal function would be of utmost value to improve quality of life. Therefore, we investigated the activity of endogenous antioxidant enzymes, oxidative stress in plasma and renal tissue, and urinary excretion of tubular proteins as candidate of early biomarkers of tubular injury during diabetes.

## 2. Methods

### 2.1. Reagents

Streptozotocin (STZ), 4-nitrophenyl-N-acetyl-*β*-D-glucosaminide, 2,4-dinitrophenylhydrazine, xanthine, nitroblue tetrazolium (NBT), xanthine oxidase, glutathione reductase (GR), and reduced glutathione (GSH) were purchased from Sigma (St. Louis, MO, USA). All other chemicals used were of the highest analytical grade available.

### 2.2. Experimental Design

All animal procedures were performed in accordance with the Mexican Federal Regulation for Animal Experimentation and Care (NOM-062-ZOO-2001) and were approved by the Bioethics and investigation Committees of the Instituto Nacional de Cardiología “Ignacio Chávez.”

Adult male Wistar rats were used at 10–14 weeks of age (250–300 g). Animals were randomly divided into three groups (*n* = 16 each group): control (C), diabetic (D), and diabetic treated with insulin (DI). Diabetes was induced by a single administration of streptozotocin (STZ) (50 mg/kg i.p.) dissolved in citrate buffer (0.1 M, pH 4.5). The control group received the same volume of citrate buffer. Blood glucose concentration was determined (Accu-Chek sensor comfort, Roche Diagnostics) 72 h after STZ administration, and only rats with glucose measurements over 20.0 mmol/L were considered diabetic for further studies.

Treatment was initiated after confirmation of diabetes. To analyze early renal changes induced by diabetes, a set of animals was sacrificed after 7 days of followup, after confirmation of diabetes (8 rats/group). The remaining rats were studied after 30 days of followup (8 rats/group). All experimental groups were maintained on laboratory diet and water* ad libitum.*

Insulin was administered i.p. (Humulin; Eli Lilly and Company, Indianapolis, IN) in an initial dose of 6 IU followed by 2 to 4 IU daily, depending on morning blood glucose values.

Rats were placed in metabolic cages (Nalgene, Rochester, NY) and urine (24 h) was collected at 7 and 30 days after diabetes induction. Urine samples were centrifuged at 5000 g for 15 minutes to remove debris, and the supernatant was vortexed and analyzed. The urinary variables measured were diuresis, glucose, creatinine clearance (IL 300 plus, clinical chemistry analyzer), proteinuria, and microalbuminuria (Albumin Rat ELISA Kit, Abcam). We evaluated the urinary excretion of NGAL, osteopontin (OPN), and NAG as biomarkers of tubular injury.

### 2.3. Evaluation of Systemic Markers of Oxidative Stress

#### 2.3.1. Preparation of the Erythrocyte Samples

At each time point (7 and 30 days), 0.5 milliliters of blood was taken from the caudal vein and collected in heparinized tubes. Blood samples were centrifuged at 1000 g for 10 min at 4°C. The upper plasma phase was carefully pipetted and transferred into Eppendorf tubes and stored at −80°C until further analysis. To prepare an erythrocyte suspension, the buffy coat on top of the erythrocyte layer was carefully removed, and the remaining erythrocytes were diluted in an isotonic NaCl solution. The suspended erythrocytes were then centrifuged at 1000 g for 10 min at 4°C, and the upper layer was removed again. Erythrocytes were washed three times and then diluted five times with ice-cold water, vortexed, and stored at −80°C until use.

#### 2.3.2. Catalase Assay

CAT activity was measured in hemolysates by a method based on the disappearance of H_2_O_2_ from a solution containing 30 mmol/L H_2_O_2_ in 10 mmol/L potassium phosphate buffer (pH 7) at 240 nm [3]. The results were expressed as U/mL.

#### 2.3.3. Glutathione Peroxidase Assay

The glutathione peroxidase (GPx) activity in erythrocyte lysates was assessed as previously described [3]. The results were expressed as U/mL.

#### 2.3.4. Superoxide Dismutase Assay

Superoxide dismutase (SOD) activity in erythrocyte lysates was measured by a competitive inhibition assay using xanthine-xanthine oxidase system to reduce NBT as previously reported [3]. Results were expressed as U/mL.

#### 2.3.5. Nitrotyrosine Measurement

Plasma protein concentrations were determined using the Bradford method [4]. Equal amounts of protein (15 *μ*g) were denatured in gel loading buffer by heating at 85°C for 5 minutes before loading onto 10% SDS-polyacrylamide gels. After electrophoretic separation, samples were transferred to polyvinylidene difluoride (PVDF) membranes and incubated at 4°C overnight with primary antibody (1 : 1000) diluted in PBST. The protein bands were visualized with enhanced chemiluminescence reagents (ECL Plus Western Blotting Detection System, Amersham Pharmacia Biotech), and analysis and intensity quantification were conducted using Kodak Electrophoresis Documentation and Analysis System 290 (EDAS 290).

### 2.4. Evaluation of Renal Markers of Oxidative Stress

#### 2.4.1. Determination of Lipid Peroxidation [4-hydroxynonenal (4-HNE)]

For the 4-HNE assay, 50 mg of kidney cortex or medulla was homogenized in ice-cold PBS; the colorimetric assay was performed in accordance to Gérard-Monnier et al. [13] and Erdelmeier et al. [14]. The results were expressed as nmol of 4-HNE/mg protein.

#### 2.4.2. Measurement of Oxidized Protein (Carbonyls Protein)

The determination of carbonyl groups in the proteins was measured using the reaction with 2,4-dinitrophenylhydrazine (DNPH) described by Levine et al. [15] with slight modifications. Protein carbonyl groups were estimated by using the molar absorption coefficient of 22, 000 M^−1^ *·* cm^−1^for DNPH derivatives, and its concentration was expressed as nmol carbonyl groups/mg protein. Guanidine solution was used as a blank.

### 2.5. Glomerular Filtration Rate

The glomerular filtration rate (GFR) was estimated by polyfructosan clearance method as previously described [16].

### 2.6. Urinary Excretion of Renal Tubule Damage Biomarkers

#### 2.6.1. Urinary Excretion of NGAL and OPN

Urinary excretion of NGAL and osteopontin (OPN) was assayed by immunoblotting. Sample volumes corresponding to 15 *μ*g of total protein were precipitated on ice for 30 min with 10% (w/v) trichloroacetic acid in PBS. Samples were then centrifuged at 13,100 g for 10 min at 4°C before washing pellets twice with ice-cold acetone. The samples were air-dried and dissolved in Laemmli buffer (62.5 mmol/L Tris-HCl (pH 6.8), 10% (v/v) glycerol, 2% (w/v) SDS, 5% (w/v) 2-mercaptoethanol, and 0.05% (w/v) bromophenol blue) followed by heating at 95°C for 5 min. Samples were loaded in SDS-PAGE gels as previously described [16]. Protein bands were visualized and intensity quantified as previously described [16]. To account for differences in hydration and urine concentration, the results were normalized to urine creatinine.

#### 2.6.2. Measurement of N-acetyl *β*-D-glucosaminidase (NAG) Activity

For the determination of NAG activity in urine samples, 4-nitrophenyl-N-acetyl-*β*-D-glucosaminide was used as substrate. One unit of enzymatic activity (U) represents the amount of enzyme, which hydrolyses one *μ*mol of substrate per min at 37°C [17, 18]. The results were expressed as U/24 h.

### 2.7. Histopathological Evaluation

To evaluate the tubular injury, the kidneys were fixed in 10% formalin in PBS and embedded in paraffin. Kidney sections (3 *μ*m) were obtained and stained with hematoxylin and eosin and later on analyzed under light microscopy (AxioPhot2 Zeiss, Germany). All slides were analyzed in a blinded fashion.

### 2.8. Statistical Analysis

Data are expressed as the mean ± SEM. Statistical differences among groups were calculated using ANOVA with Bonferroni correction (Prism 4.0; GraphPad Software, San Diego, CA, USA). Significance for all statistical comparisons was set at *P* < 0.05.

## 3. Results 

### 3.1. Physiologic Characteristics

After 7 days of followup, diabetic rats were hyperglycemic and glycosuric compared to control. Insulin treatment prevented these changes (Table 1). There were no significant differences between experimental groups in proteinuria, albuminuria, serum creatinine, urinary creatinine, or creatinine clearance. Because renal functional changes were imperceptible at this early time point, we did not measure GFR.

After 30 days, diabetic rats were hyperglycemic and glycosuric, with high diuresis, proteinuria, albuminuria, and low body weight compared to the control group and diabetic rats treated with insulin (Table 1). Furthermore, creatinine clearance was increased in the diabetic rats compared to the control group, suggesting glomerular hyperfiltration. This well-known effect induced by diabetes was demonstrated by a significant increase in polyfructosan clearance. Insulin treatment effectively prevented glomerular hyperfiltration in diabetic rats. Additionally, body weight, serum creatinine, diuresis, urinary creatinine, proteinuria, albuminuria, and blood glucose levels were not statistically different between diabetic insulin-treated and control group (Table 1).

### 3.2. Evaluation of Markers of Systemic Oxidative Stress

**Figure 1:**
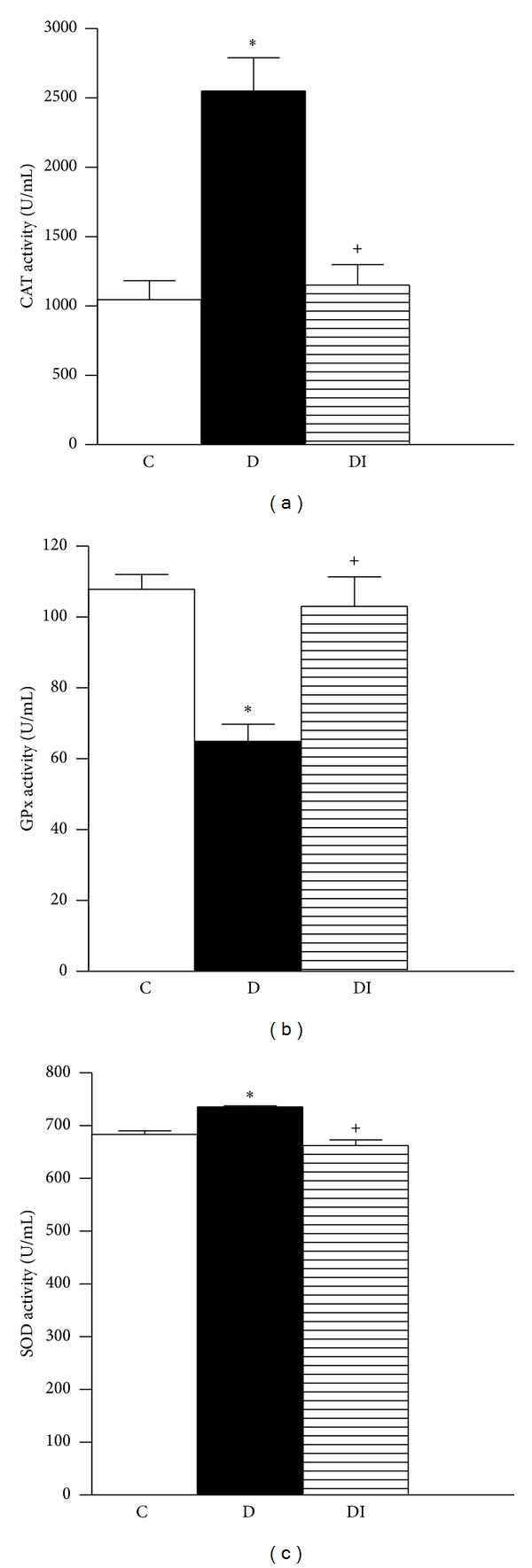
Activity of antioxidant enzymes in erythrocyte lysates 30 days after diabetes induction. (a) Catalase, (b) glutathione peroxidase, and (c) superoxide dismutase. C: control, D: diabetic, and DI: diabetic insulin treated. Data are mean ± SEM of eight animals in each group *P < 0.05 versus C; ^+^ *P* < 0.05 versus D.

**Figure 2:**
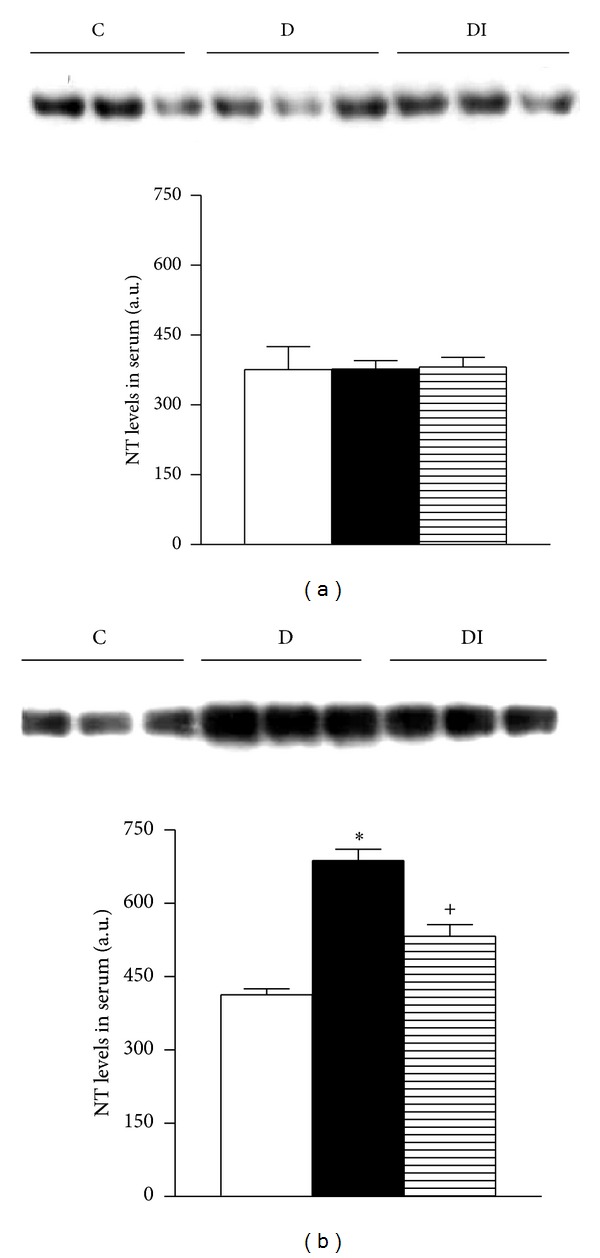
Immunoblot and semiquantitative analysis of nitrotyrosine levels in plasma. (a) 7 days; (b) 30 days. C: control, D: diabetic, and DI: diabetic insulin treated. Data are mean ± SEM of eight animals in each group. *P < 0.05 versus C; ^+^ *P* < 0.05 versus D.

Hyperglycemia-induced oxidative stress was assessed by measuring the activity of antioxidant enzymes CAT, GPx, and SOD in erythrocyte lysates and by determination of serum nitrotyrosine levels using western blot analysis. At 7 days, there were no statistical differences in the activities of CAT, GPX, and SOD (Table 2) between diabetic and control rats. However, after 30 days, CAT and SOD activity was significantly increased in diabetic rats (Figures 1(a) and 1(c) resp.), while GPx activity was decreased (Figure 1(b)). These alterations in antioxidant enzyme activity were associated with a significant increase in nitrotyrosine levels in diabetic rats (Figures 2(a) and 2(b)). However, insulin treatment preserved normal enzymatic activity and nitrotyrosine levels.

### 3.3. Evaluation of Renal Markers of Oxidative Stress

**Figure 3:**
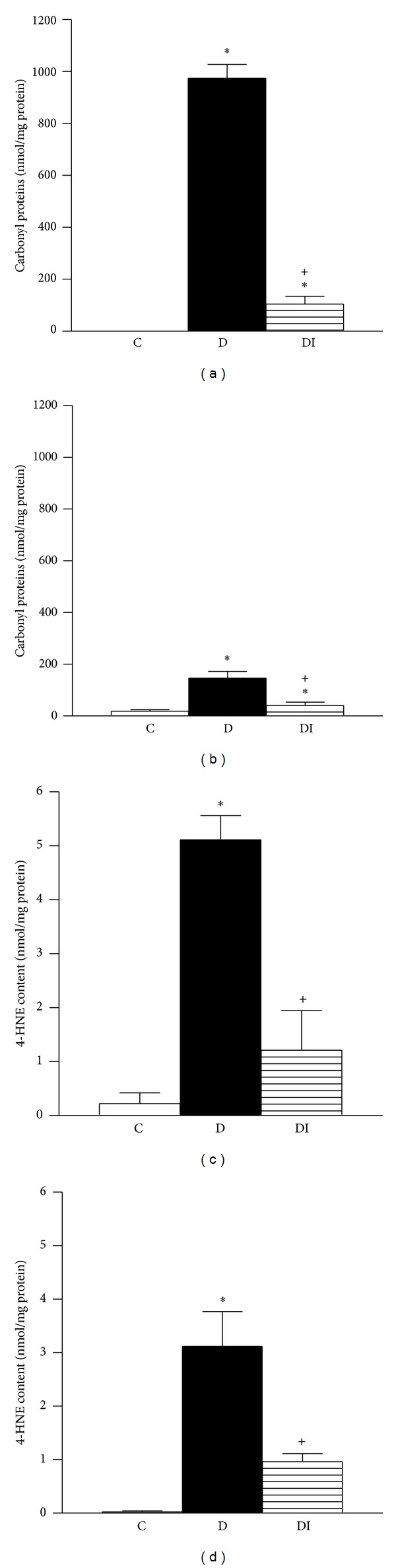
Oxidative stress in kidney tissue from animals with 30 days after diabetes induction. Oxidized proteins in (a) cortex and (b) medulla; 4-hydroxynonenal content in (c) cortex and (d) medulla. C: control, D: diabetic, and DI: diabetic insulin treated. Data are mean ± SEM of eight animals in each group. *P < 0.05 versus C; ^+^ *P* < 0.05 versus D.

To assess local oxidative stress, we evaluated lipid and protein oxidation by measuring tissue content of 4-hydroxynonenal (4-HNE) and carbonyl proteins. Lipid and protein oxidation were similar among all experimental groups in the renal cortex and medulla after 7 days of the diabetes induction (Table 2). However, 4-HNE and carbonyl protein content in renal cortex and medulla were significantly increased in diabetic rats compared to the control group after 30 days (Figure 3). This diabetes-induced renal oxidative stress was prevented by insulin treatment (Figure 3).

Together, these findings suggest that during diabetes systemic and renal oxidative stress are a secondary effect of hyperglycemia.

### 3.4. Urinary Excretion of Tubular Damage Biomarkers

**Figure 4:**
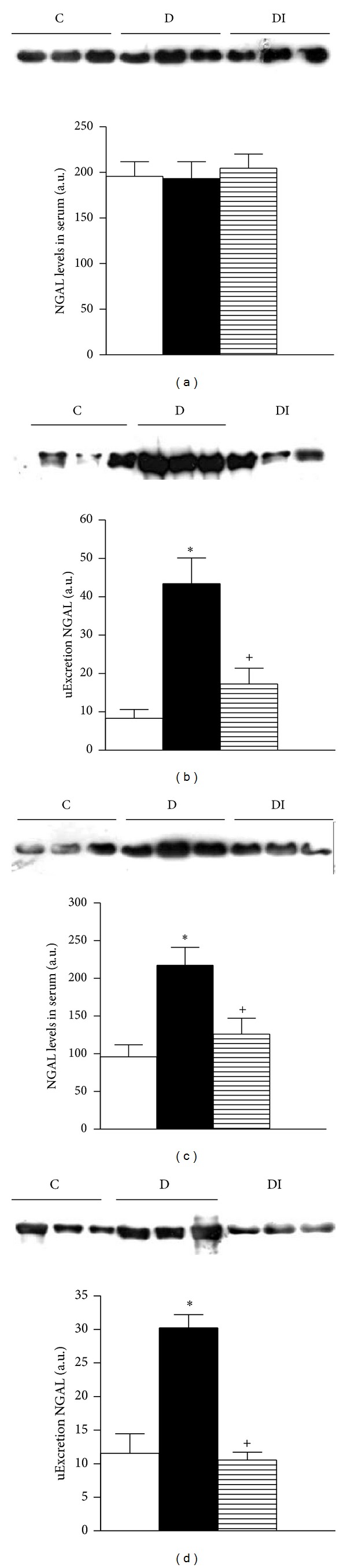
Immunoblot and semiquantitative analysis of NGAL levels. 7 days (a) and (b); 30 days (c) and (d). Plasma (a) and (c) and urine (b) and (d). C: control, D: diabetic, and DI: diabetic insulin treated. Data are mean ± SEM of eight animals in each group. *P < 0.05 versus C; *P < 0.05 versus D.

**Figure 5:**
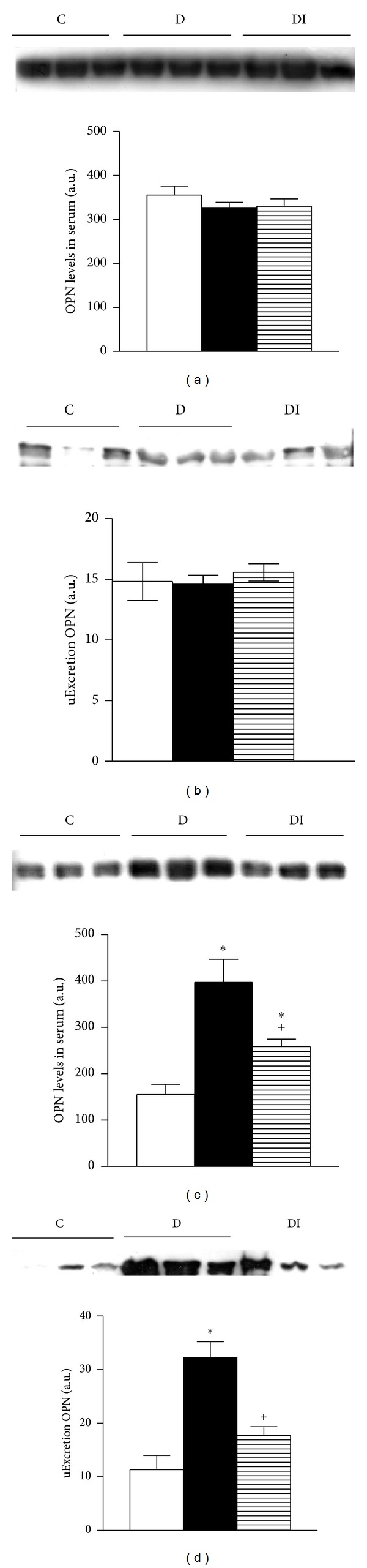
Immunoblot and semiquantitative analysis of OPN levels. 7 days after diabetes induction (a) and (b); 30 days after diabetes induction (c) and (d). Plasma (a) and (c) and urine (b) and (d). C: control, D: diabetic, and DI: diabetic insulin treated. Data are mean ± SEM of eight animals in each group. *P < 0.05 versus C; ^+^ *P* < 0.05 versus D.

Seven days after induction of diabetes, there were no changes in serum expression of NGAL and OPN (Figures 4(a) and 5(a)). In contrast, urinary excretion of uNGAL was significantly higher in diabetic rats compared to healthy controls and diabetic insulin-treated animals (Figure 5(b)). The other evaluated biomarkers (OPN and NAG) did not differ among the studied groups.

**Figure 6:**
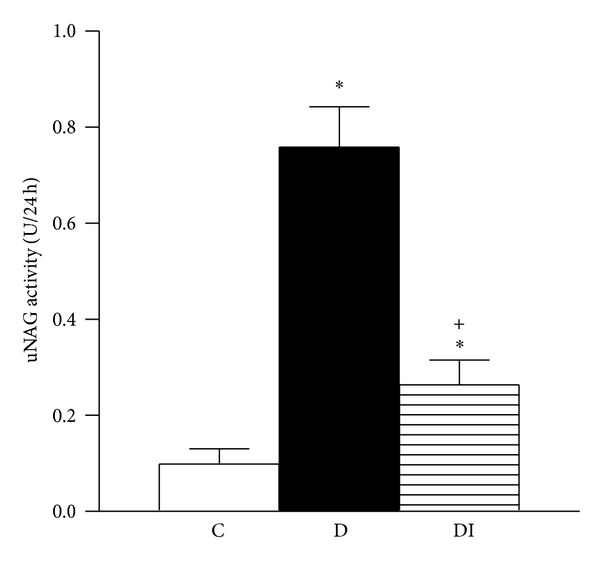
Urinary excretion of NAG. C: control, D: diabetic, and DI: diabetic insulin treated. Data are mean ± SEM of eight animals in each group *P < 0.05 versus C; ^+^ *P* < 0.05 versus D.

Thirty days after diabetes induction, NGAL and OPN serum levels were higher in serum from the diabetic group compared to the control group (Figures 4(c) and 5(c)). These results were associated with increased urinary excretion of NGAL and OPN in diabetic rats (Figures 4(d) and 5(d)). Moreover NAG urinary excretion was increased in diabetic rats compared to control rats (Figure 6). This increase in urinary excretion of tubular proteins was prevented by insulin treatment.

### 3.5. Histological Analysis

**Figure 7:**
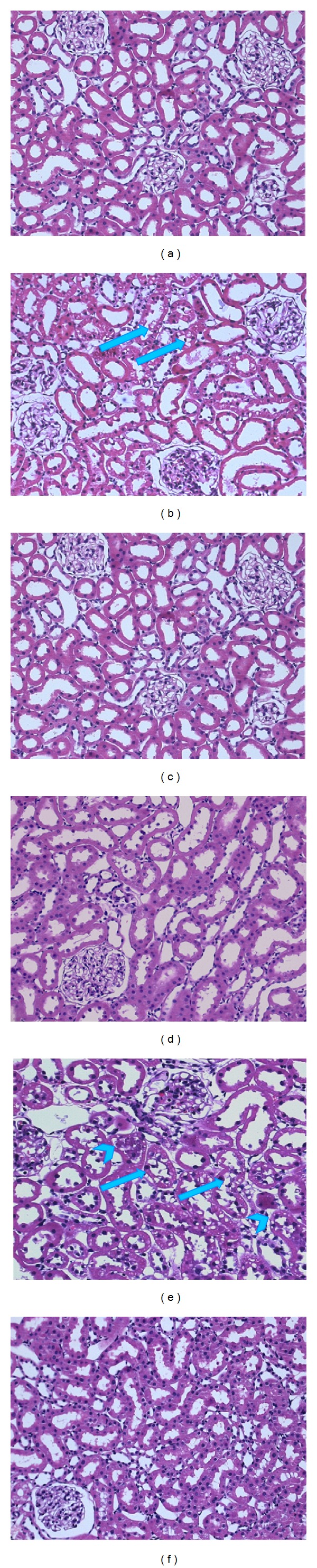
(a)–(f) are representative microphotographs of cortical kidney sections stained with hematoxylin/eosin. (a)–(c) From animals sacrificed 7 days after diabetes induction and (d)–(f) from animals euthanized 30 days after induction. (a) and (d) Kidneys from control rats show no lesions. (b) Diabetic animal (7 days) exhibits tubular epithelium vacuolar degeneration (arrows). (e) Diabetic rat (30 days) shows vacuolization (arrows), detachment, and debris of the tubular epithelial cells in the tubular lumen (arrowheads). (c) and (f) Diabetic insulin treated rats, with no abnormalities and considerable attenuation of these changes, respectively. (200x original magnification).

Histological analysis of the kidney from control rats revealed normal glomeruli and proximal and distal convoluted tubules 7 days after diabetes induction (Figure 7(a)). The kidney of diabetic rats showed vacuolar degeneration of the tubular epithelium (Figure 7(b)); these changes were absent in diabetic insulin-treated rats (Figure 7(c)). Thirty days after diabetes induction, epithelial tubular cells showed vacuolization and cell detachment, with cellular debris detected in the tubular lumen (Figure 7(e)). Insulin treatment in diabetic rats greatly attenuated these lesions (Figure 7(f)).

In summary, insulin treatment in diabetic rats significantly blocked the increase in oxidative stress and urinary excretion of tubular injury markers.

## 4. Discussion

Diabetic nephropathy is one of the leading causes of chronic kidney diseases worldwide. It is therefore of utmost importance to find early markers for diabetes-associated renal disease in order to provide prompt therapeutic interventions that retard progression in patients at risk for developing chronic disease. Therefore, the aim of this study was to evaluate if markers of oxidative stress or urinary biomarkers of renal damage might be useful tools to detect early signs of renal diabetic damage.

Diabetic nephropathy has traditionally been considered as a glomerular disease; however, it is now widely accepted that the rate of deterioration in renal function correlates best with the degree of tubulointerstitial damage [19]. In fact, tubular involvement may occur before glomerular damage develops; several tubular proteins and low molecular weight enzymes are detectable in urine before the appearance of microalbuminuria and the rise in SrC [5–7, 20]. Currently, the most frequently evaluated urinary enzymes include NAG, NGAL, and KIM-1 [6, 7, 17, 20].

To investigate oxidative stress and urinary excretion of tubular markers, we used diabetic rats at 7 and 30 days after diabetes induction. At 7 days after induction, the diabetic rats were hyperglycemic but did not show any alternations in oxidative stress markers. However, at 30 days after induction the diabetic rats presented with diuresis, glycosuria, proteinuria, albuminuria, and glomerular hyperfiltration. Glomerular hyperfiltration and proximal tubular hyperreabsorption are among the distinctive features of early diabetic nephropathy [21]. Additionally, systemic oxidative stress data was characterized by a significant increase in CAT and SOD activity and 3-NT levels; in contrast, GPx activity was decreased. Tissue oxidative stress was demonstrated by a high renal content of 4-HNE and carbonyl proteins in diabetic rats. These data are consistent with other studies that report oxidative stress in rats and patients with hyperglycemia, supporting the hypothesis that oxidative stress is a general pathophysiological pathway in the development of diabetic nephropathy [3, 22–25].

In the present study, glomerular hyperfiltration was associated with renal oxidative stress. There is no definitive mechanism for the role of oxidative stress in the induction of hyperfiltration; however, some hypotheses imply that increased mitochondrial superoxide production is a major mechanism of microvascular damage in diabetes [26]. Structural alterations in afferent and efferent arterioles may partially explain the functional changes that lead to glomerular hyperfiltration, as has been observed in other models [27].

Diabetes-stimulated renal damage was detectable in urinary excretion of biomarkers. A significant increase in NGAL excretion was apparent by 7 and 30 days after diabetes induction. Upregulation of NGAL, which was detected in urine but not in plasma, suggests that NGAL is produced primarily in renal tissue, specifically in the tubule [22]. Our data suggest that the early increase in urinary excretion of NGAL is likely dependent on hyperglycemia rather than oxidative stress. In order to confirm these results, we administered the antioxidant Tempol (15 mg/kg/day) to diabetic rats during 7 days. Treatment with Tempol did not modify urinary excretion of NGAL in diabetic animals (data not shown). Therefore, these results support the notion that the early increase in urine NGAL is not dependent on increased oxidative stress. Thirty days after diabetes induction, NGAL and OPN levels were increased in both the serum and urine of diabetic rats. These data suggested that tubular injury had occurred. This was supported by the epithelial tubular damage observed histologically and characterized by vacuolar degeneration and the presence of detached cells and debris in the tubule lumen. These results suggested that tubular injury was mediated by the diabetogenic environment and, importantly, that urinary excretion of OPN, NAG, and in particular NGAL may be early detected markers for renal damage during diabetes.

Other studies have reported increases in urinary excretion of kidney injury markers, including lipocalin 2 (Lcn-2), OPN, α-glutathione S-transferase (α-Gst), *μ*-glutathione S-transferase (*μ*-Gst), and beta-2 microglobulin (*β*2m) in diabetes [22, 28] and ischemic or nephrotoxic injury in both animals and humans [11, 12, 28]. However, to our knowledge this is the first study evaluating the temporal expression pattern of urinary biomarkers NGAL, OPN, and NAG in early experimental diabetes. Serum NGAL has been described as a sensitive and specific biomarker for early identification of kidney injury following cardiac surgery [11], for CKD in children [12] and for diabetic patients with and without microalbuminuria [7, 29–31].

It has also been reported that OPN levels inversely correlate with decreased glomerular filtration rate, severity of nephropathy, and coronary artery disease during the progression of human and experimental diabetes [32–34]. Urinary NAG is known to be distributed more widely in the nephron and released as a result of tubular damage [35]. Our study showed that the uNAG was significantly higher in diabetic groups and was associated with duration of diabetes. These data are consistent with other reports, which state that excretion of NAG indicates proximal tubular dysfunction [7, 22, 36, 37].

Tubular cell injury impairs reabsorption, which in turn leads to increased excretion of these nonreabsorbed proteins. Thus, the increased excretion rates observed in our study reflect tubular cell damage.

## 5. Conclusions

The appearance of NGAL in urine is associated with hyperglycemia and precedes the development of oxidative stress and the appearance of other urinary markers or elevations in serum creatinine, albuminuria, OPN, and NAG. This suggests that tubular damage may precede glomerular injury. Our results suggest that NGAL, OPN, and NAG might be used as early, sensitive, and noninvasive urinary biomarkers of renal injury. More studies are needed to confirm our results and to detect the underlying mechanisms of tubular injury in diabetic nephropathy.

## Figures and Tables

**Table 1:** Biochemical and physical characteristics of experimental groups.

	Control		Diabetic		Diabetic insulin treated	
	7 days	30 days	7 days	30 days	7 days	30 days
Body weight (g)	259 ± 3.34	416.0 ± 8.13	279 ± 3.08	263.5 ± 7.28∗	269 ± 5.662	372.8 ± 16.59^+^
Blood glucose (mg/dL)	91.16 ± 5.04	91.75 ± 4.27	402.12 ± 21.47∗	402.11 ± 24.61∗	101.2 ± 7.002^+^	102.8 ± 5.787^+^
Serum creatinine (mg/dL)	0.45 ± 0.03	0.47 ± 0.08	0.48 ± 0.05	0.39 ± 0.09	0.42 ± 0.02	0.41 ± 0.02
Diuresis (mL/24 hrs)	14.45 ± 1.47	16.3 ± 4.3	22.82 ± 3.72∗	37.26 ± 3.55∗	16 ± 1.3^+^	16.69 ± 2.8^+^
Glycosuria (mg/dL)	1.06 ± 0.06	0 ± 0	4797.1 ± 148.4∗	4605.55 ± 130.94∗	2.50 ± 1.12^+^	6.2 ± 2.44^+^
Urine creatinine (mg/mL/24 hrs)	6.26 ± 0.33	6.52 ± 1.56	8.21 ± 0.61	9.41 ± 0.59∗	4.87 ± 0.72	5.72 ± 1.21^+^
Proteinuria (mg/24 hrs)	19.36 ± 1.79	14.43 ± 1.51	25.87 ± 2.51	37.26 ± 3.55∗	17.4 ± 1.29	11.62 ± 3.97^+^
Glomerular filtration rate (mL/min)	ND	1.39 ± 0.10	ND	3.519 ± 0.46∗	ND	1.725 ± 0.25^+^
Albuminuria (*μ*g/24 hrs)	206 ± 23.42	189.4 ± 26.61	252 ± 40.19	446.6 ± 55.24∗	247.2 ± 19.63	178.6 ± 23.92^+^
Creatinine clearance (mL/min)	0.99 ± 0.09	1.02 ± 0.18	0.99 ± 0.08	1.87 ± 0.3∗	0.91 ± 0.16	0.79 ± 0.17^+^

ND: not determined. Data are mean ± SEM of 8 animals in each group. *P < 0.05 versus control; ^+^ *P* < 0.05 versus diabetic.

**Table 2:** Evaluation of oxidative stress at 7 days.

	Control	Diabetic	Diabetic-insulin treated
CAT (U/mL)	1780 ± 294.7	1459 ± 109.6	1791 ± 205.3
GPx (U/mL)	106.2 ± 1.19	116.1 ± 2.46	109.1 ± 3.17
SOD (U/mL)	651.8 ± 9.52	662.0 ± 10.96	683.6 ± 6.79
Carbonyl content in Ctx (nmol/mg protein)	1.75 ± 0.90	4.36 ± 1.67	2.72 ± 0.25
Carbonyl content in Med (nmol/mg protein)	13.73 ± 3.01	7.91 ± 2.28	7.26 ± 2.89
4-HNE content in Ctx (nmol/mg protein)	0.22 ± 0.19	0.89 ± 0.22	0.83 ± 0.27
4-HNE content in Med (nmol/mg protein)	0.41 ± 0.25	1.09 ± 0.28	1.04 ± 0.30

CAT: catalase, GPx: glutathione peroxidase, SOD: superoxide dismutase, Ctx: renal cortex, Med: renal medulla, and 4-HNE: 4-hydroxynonenal. Data are mean SEM of 8 animals in each group. *P* < 0.05 versus control; *P* < 0.05 versus diabetic.
